# Three Cases of Amputation of the Penis

**Published:** 1851-07

**Authors:** Frank H. Hamilton

**Affiliations:** One of the Surgeons of the Buffalo Hospital of the Sisters of Charity


ART. IV.—*Three Cases of* AMPUTATION OF THE PENIS. Reported by FRANK H. HAMILTON, one of the Surgeons of the Buffalo Hospital of the Sisters of Charity.

*Case* 1.—J. D., aged 60, admitted to Hospital Nov. 2d, 1850. Never had venereal disease; habits temperate. One year since, blenorrhœa urethræ commenced, which has continued in some degree until now. Six months since, the penis was injured by a bruise, and from this time it has steadily increased in size.

Prepuce is now enormously enlarged, and is covered upon its inner surface with small granulations like millet seeds. It has also ulcerated at points. The glans is completely involved in a scirrhus disease.

*Operation.*—The patient being completely narcotized with chloroform, I proceeded to amputate the penis, back of the glans, with a common scalpel. Four arteries required the ligature. I closed the ends of the prepuce with two sutures, in order to prevent retraction, and then dressed with a roller and lint. I pursued the same course with each of the following cases.

In this operation I introduced, before the incisions were commenced, a silver catheter, into the bladder, and permitted it to remain until the operation was completed. I thus avoided the difficulty which sometimes attends the introduction of a catheter after the operation.

I removed the catheter a few days after, and did not re-introduce it. The wound healed slowly, but without accident.

*Case* 2.—Mr. L. ———, aged 55. Health rather feeble; had a stricture many years; phymosis from birth; habits perfectly temperate; was never exposed to venereal disease. The glans penis and prepuce are now enlarged indurated and ulcerated. The prepuce is the part chiefly implicated, and is scirrhus. He says when it began to enlarge about two years since, the granulations looked like homœopathic pills.

*Operation.*—Nov. 5, 1850. Present Messrs. Nichols, and Boardman; I amputated the entire prepuce and a portion of the glans. Patient completely under the influence of chloroform. Four arteries tied. A silver catheter was then introduced, and it was retained in place a day or two, after which it was removed, and was occasionally introduced. The wound healed rapidly.

*Case* 3.—C. G., aged 45; feeble; habits temperate; never exposed to venereal disease. Nine months ago he felt an itching on the prepuce; it then became swollen. It is now very large, indurated, ulcerated and painful. The disease has involved the glans, and is of a scirrhus character.

*Operation.*—May 2, 1851.—Present Drs. Smith, Adams, and Allen. Having produced nearly complete anæsthesia with chloroform, I amputated below the glans. Six arteries required the ligature. A flexible catheter was then introduced, and kept in place a day or two. The wound has healed kindly.

